# Factors associated with casual sexual behavior among college students in Zhejiang Province, China: A cross-sectional survey

**DOI:** 10.1371/journal.pone.0304804

**Published:** 2024-07-12

**Authors:** Zhongrong Yang, Wanjun Chen, Weiyong Chen, Qiaoqin Ma, Hui Wang, Tingting Jiang, Yun Fu, Xin Zhou

**Affiliations:** 1 Huzhou Center for Disease Control and Prevention, Huzhou, Zhejiang province, China; 2 Department of HIV/STD Control and Prevention, Zhejiang Provincial Center for Disease Control and Prevention, Hangzhou, Zhejiang Province, China; Universita Cattolica del Sacro Cuore Sede di Roma, ITALY

## Abstract

This study aimed to analyze the characteristics and risk factors associated with casual sexual behavior among sexually active college students and to contribute to AIDS prevention and control efforts among this demographic. A cross-sectional survey was conducted using a stratified cluster sampling technique. Self-reported sexually active college students were selected as respondents from 11 cities in Zhejiang Province from October 8 to November 30, 2018. A questionnaire was used to collect variables such as demographic information, sexual attitudes, intervention acceptance, and self-efficacy of condom use. Univariate and multivariate analyses were performed. Among 3,771 college students who reported engaging in sexual activity, 675 (17.90%) reported engaging in casual sexual encounters. The multivariate analysis revealed multiple factors associated with casual sexual behavior among students: being male, originating from a city/town, having pursued HIV testing education in the last year, seeking HIV risk self-assessment within the last year, accepting to engage in one-night stand behavior, accepting to partake in commercial sexual activity, having conducted HIV antibody tests within the last year, homosexual partner or homosexual/heterosexual partner, demonstrating self-efficacy in condom usage, and monthly living expenses falling within the range of 1001–1500 yuan. Additionally, students with knowledge that appearance does not determine HIV infection, a proclivity for seeking HIV counseling and testing following high-risk sexual behavior and awareness that the centers for disease control provides HIV diagnosis were found to have significant associations with casual sexual activity. Casual sex is significantly prevalent among college students, with male, students from urban areas, those who accepted to engage in one-night stand behavior and partook in commercial sexual activity demonstrating a higher propensity for such behavior. This tendency can be attributed to several factors including a more liberal sexual attitude, a rudimentary understanding of HIV risk, and a low adoption rate of HIV testing. Therefore, it is imperative to enhance HIV prevention and education among college students.

## Introduction

Antiretroviral therapy for AIDS has the potential to curtail viral replication, enhance immunological self-repair, and prevent additional damage [1,2]. Nevertheless, the number of new HIV infections reported globally in recent years has remained high, suggesting the continued severity of the AIDS epidemic, which has become a global health burden [3].The Joint United Nations Programme on HIV/AIDS has passed the Sustainable Development Goals to end the spread of HIV/AIDS by 2030 [4]. University students, as a vast cohort of young people, have become a group susceptible to infection because their engaging in high-risk behaviors. AIDS-related deaths are among the primary causes of death in this group [5]. In recent years, the number of Chinese students infected with HIV/AIDS has risen rapidly, with approximately 3,000 cases reported annually, making them a key group in HIV/AIDS prevention efforts. Given that young students are at a stage of sexual activity, the pathway for transmission leading to infection is primarily sexual, rendering this a pressing public health issue and social concern [6,7].

The transmission routes of HIV are diverse, and students’ levels of awareness and preventive consciousness of the disease directly affect its spread rate and scope [8]. By widely disseminating preventive knowledge about HIV, including transmission routes, prevention methods, and risk factors, it can enhance students’ awareness and preventive consciousness, thereby reducing the risk of HIV infection [9,10]. At the same time, promoting the use of condoms is considered an effective way to prevent sexually transmitted diseases (STDs) among students, as it is widely regarded as a simple, inexpensive, and highly effective preventive measure [11]. Correct condom use can effectively reduce the risk of HIV and other STDs [12]. Therefore, gaining an in-depth understanding of condom use, challenges, and strategies to promote correct use is of great importance for raising students’ health awareness and preventing the spread of HIV and other STDs.

Previous research indicates that there is a continuous increase in the proportion of high-risk behaviors such as casual sex, commercial sex, and pre-sexual drinking. This likely leads to an increase in STDs and unplanned pregnancies among college students, resulting in substantial harm to their physical and mental health [13,14]. The rapid rise in the HIV infection rate among Chinese university students has drawn national attention. Since 2015, the National Health Commission and the Ministry of Education have jointly issued documents to prevent HIV infection and have implemented HIV prevention education and behavioral interventions in universities. Across the country, there is a greater focus on homosexual behavior among men, with less attention paid to casual sexual acts among heterosexual university students [6]. Concurrently, heterosexual university students’ participation in homosexual activities should be noted, as this may considerably impact the spread of HIV [15].

Presently, Chinese university students tend to adopt a more tolerant attitude toward premarital sexual behavior, which can foster the habit of engaging in risky sexual activities without appropriately using contraceptives [16]. Therefore, understanding the characteristics and factors influencing sporadic sexual activity among students is of vital importance in devising targeted prevention strategies for the spread of HIV/AIDS. Our large-scale cross-sectional survey aimed to analyze the distinctive features and influencing factors of students’ casual sexual behaviors, providing an evidence-based foundation for universities to develop targeted HIV/AIDS prevention strategies.

## Materials and methods

### Study design and procedure

We employed a cross-sectional survey approach. Students on the school premises completed electronic surveys, administered by classroom teachers by scanning a QR code on their mobile phones. Conversely, students undertaking off-site internships were sent a link to the survey, with clear instructions prompting them to complete it independently.

The survey was conducted by staff members from local disease prevention and control centers and class advisors from various universities who had undergone uniform training before using a standard questionnaire for an anonymous survey. Prior to the survey, the investigators explained the purpose, significance, methodology, and privacy policy to the participants, which were also included in the questionnaire preamble. Participants were informed that the purpose of the survey was to develop an AIDS/STDs prevention strategy and that the survey was anonymous and only analyzed group data, not individual data. This study was reviewed and approved by the Ethics Committee of the Zhejiang provincial center for disease control and prevention (approval number 2018–036). All the participants provided written informed consent.

### Participants and settings

A survey was conducted with university students from 13 colleges and universities in 11 cities of Zhejiang Province from October 8 to November 30, 2018, including three in Hangzhou City and one in each of the remaining ten cities, chosen through recommendations by local disease control centers. This cross-sectional, province-wide survey included 31,674 students. A stratified cluster sampling method was employed. First, a random digit table was used to select three departments from each university, which were then divided into four strata based on academic years 1–4. Within each stratum, classes were selected using a random digit table, with 1,241 classes in total.

The survey comprised 14,320 male and 17,354 female university students. Of these, 3,771(11.91%) self-reported sexual activity and disclosed their sexual partners. A total of 675 students reported casual sexual behaviors, comprising 2.13% of the overall student count. Only students who reported sexual activity and disclosed their sexual partners were included in the study. A comparison of basic characteristics between the excluded and included students revealed no statistical difference.

### Variables and measurement

The questionnaire was developed based on existing literature reviews, research team discussions, and a pilot study conducted among students in a school class. The survey addressed the general sociological characteristics of the participants, the knowledge related HIV transmission, sexual attitudes, intervention acceptance, and confidence in using condoms. The knowledge-based questions in the survey numbered 1–3 were as follows: “The main mode of HIV transmission among students in China is through homosexual behavior or not?” (the correct answer is “Yes”), “Is it possible to determine if a person is infected with HIV through their appearance?”(the correct answer is “No”), and “Should one proactively seek HIV counseling and testing after having high-risk sexual behavior?”(the correct answer is “Yes”) [17]. The sexual attitudes included two variables:“whether accept to engage in one-night stand behavior(the measurement was accept, don’t accept, don’t know)” and “whether accept to partake in commercial sexual activity(the measurement was accept, don’t accept, don’t know)”.Casual sexual behavior pertains to the sexual activities with lover/husband or wife engaged in by an individual over the past year, encompassing casual encounters, such as one-night stands, as well as sexual interactions with acquaintances.

The efficacy of condom use is gauged by three aspects: your confidence in discussing condom use with your sexual partner before engaging in sexual activities, your determination to abstain from sex when your partner refuses to use or does not carry a condom, and your assurance of preparing a condom before sexual activities. Each question offers five options: very confident, confident, somewhat confident, not confident, and not at all confident, scored from 3 to -1, respectively. The scores were categorized into three groups: highly confident (9 points), confident (5–8 points), and not confident (less than 4 points) [18]. The test had a Cronbach’s alpha coefficient of 0.778.

### Data analysis

Data analysis was conducted using SPSS software (version 21.0; SPSS Inc., Chicago, IL, USA). Various factors such as age, sex, household registration, hometown, monthly living expenses, attitudes toward sexual behavior, preventative knowledge, and characteristics, etc. were demonstrated using constituent ratios or rates. A χ^2^ test was used to compare the demographic characteristics of college students who had engaged in casual sexual behavior. The independent variables included demographic characteristics, attitudes toward sex, preventative knowledge, intervention acceptance, and self-efficacy in condom use. Single-factor logistic regression was used to analyze the factors influencing casual sexual behavior among college students. With the occurrence of casual sexual behavior serving as the dependent variable, variables with P<0.1 were included in the model. Multivariate logistic regression analysis was then conducted, with the significance level set at P<0.05 to determine statistical significance.

## Results

### Participants characteristics

Among the 3,771 sexually active university students, 675 engaged in casual sexual behaviors, accounting for 17.9%. The average age of students involved in casual sexual practices was 20.10 ± 1.528, while that of those uninvolved was 20.20 ± 1.331. Statistically significant differences existed between the two groups regarding age, sex, hometown origin, and monthly living expenses (P<0.05). However, no statistically significant differences existed based on whether the students were inside or outside the province (Table 1).

**Table 1:** Univariate analysis of associated factors with casual sexual behavior among participants.

Variables	Casual sexual behavior group (n = 675, %)		Non-casual sexual behavior group (n = 3096, %)		*χ* ^*2*^	*P*
**Age (yrs)**						
≤19	233 (20.3)		914 (79.7)		6.562	0.038
20–21	342 (16.8)		1696 (83.2)			
≥22	100 (17.1)		486 (82.9)			
**Gender**					97.770	<0.001
Female	92 (8.3)		1014 (91.7)			
Male	583 (21.9)		2082 (78.1)			
**Province of household registration** *					0.120	0.729
Zhejiang Province	491 (18.0)		2235 (82.0)			
Other provinces	183 (17.5)		861 (82.5)			
**Source of origin hometown**					20.881	<0.001
Rural area	357 (21.4)		1931 (78.6)			
Town/city	318 (15.6)		1165 (84.4)			
**Monthly living expenses (Yuan, CNY** ^**#**^ **)**					36.922	<0.001
≦1000	209 (22.7)		711 (77.3)			
1001–1500	203 (13.5)		1302 (86.5)			
≧1501	263 (19.5)		1083 (80.5)			
**The main mode of HIV transmission among students in China is through homosexual behavior or not?**					3.612	0.057
Error/Unknown	347 (16.8)		1716 (83.2)			
Correct	328 (19.2)		1380 (80.8)			
**Is it possible to determine if a person is infected with HIV through their appearance?**					122.277	<0.001
Error/Unknown	176 (35.7)		317 (64.3)			
Correct	499 (15.2)		2779 (84.8)			
**Should one proactively seek HIV counseling and testing after having high-risk sexual behavior?**					63.385	<0.001
Error/Unknown	67 (41.4)		95 (58.6)			
Correct	608 (16.8)		3001 (83.2)			
**Whether accepted the school’s publicity on AIDS testing in the last year**					28.325	<0.001
No	221 (14.0)		1359(86.0)			
Yes	454 (20.7)		1737(79.3)			
**Whether accepted the AIDS risk self-assessment carried out by the school**					93.676	<0.001
No	345 (13.7)		2181(86.3)			
Yes	330 (26.5)		915(73.5)			
**Whether accept to engage in one night stand behavior**					362.777	<0.001
Don’t accept/don’t know	167(7.7)		2004(82.3)			
Yes	508(31.8)		1092(68.2)			
**Whether accept to partake in commercial sexual activity**					353.649	<0.001
Don’t accept/don’t know	314 (11.1)		2512 (88.9)			
Yes	361 (38.2)		584 (61.8)			
**Sexual partners**					218.801	<0.001
Heterosexual	521 (15.1)		2931 (84.9)			
Homosexual/Both	154 (48.3)		165 (51.7)			
**Whether have been tested for HIV**					31.407	<0.001
No	563 (16.7)		2809(83.3)			
Yes	112 (28.1)		287(71.9)			
**Whether known that the CDC provides HIV testing**					19.209	<0.001
No	98 (26.1)		277(73.9)			
Yes	577 (17.0)		2819(83.0)			
**Demonstration of self-efficacy in condom usage** ^*^					29.888	<0.001
4 scores and below	160 (14.2)		970(85.8)			
5–8 scores	197 (15.5)		1074(84.5)			
9 scores	283(21.9)		1008(78.1)			

^*^There is missing data.

^***#***^*CNY*, *Chinese yuan*.

### Factors associated with casual sexual behavior among college students

The results of the univariate analysis of all variables are presented in Table 1. Variables in the univariate analysis (P<0.1) were incorporated into the model for multivariate logistic analysis. Multivariate analysis (Table 2) showed that being male (adjusted odds ratio [AOR] = 1.69, 95% confidence interval [95% CI] = 1.28–2.23), originating from a city/town (AOR = 1.28, 95% CI = 1.05–1.57), having pursued HIV testing education in the last year (AOR = 1.26, 95% CI = 1.01–1.58), seeking HIV risk self-assessment within the last year (AOR = 1.64, 95% CI = 1.32–2.04), accepting to engage in one night stand behavior (AOR = 3.23, 95% CI = 2.53–4.12), accepting to partake in commercial sexual activity (AOR = 1.92, 95% CI = 1.53–2.42), having conducted HIV antibody tests within the last year (AOR = 1.36, 95% CI = 1.02–1.82), homosexual partner or homosexual/heterosexual partner (AOR = 3.43, 95% CI = 2.57–4.58),demonstrating self-efficacy in condom usage of 9 scores (AOR = 1.28, 95% CI = 1.00–1.64)were risk factors associated with casual sexual activity among participants. Multivariate analysis also indicated monthly living expenses falling within 1001–1500 yuan (AOR = 0.74, 95% CI = 0.57–0.95), students with knowledge that appearance does not determine HIV infection (AOR = 0.55, 95% CI = 0.42–0.70), a proclivity for seeking HIV counseling and testing following high-risk sexual behavior (AOR = 0.60, 95% CI = 0.40–0.90) and awareness that the centers for disease control(CDC) provides HIV diagnosis (AOR = 0.66, 95% CI = 0.49–0.90) were protected factors associated with casual sexual activity among participants.

**Table 2:** Analysis of associated factors with casual sexual behavior among participants.

Variables	*AOR(95% CI)*	*P*
**Age (yrs)**		
≤19	*Ref*.	
20–21	0.88(0.71–1.10)	0.259
≥22	0.81(0.60–1.10)	0.173
**Gender**		
Female	*Ref*.	
Male	1.69(1.28–2.23)	<0.001
**Source of origin hometown**		
Rural area	*Ref*.	
Town/city	1.28(1.05–1.57)	0.017
**Monthly living expenses (Yuan, CNY** ^**#**^ **)**		
≦1000	*Ref*.	
1001–1500	0.74(0.57–0.95)	0.019
≧1501	0.88(0.69–1.13)	0.344
**The main mode of HIV transmission among students in China is through homosexual behavior or not?**		
Error/Unknown	*Ref*.	
Correct	1.01(0.83–1.23)	0.939
**Is it possible to determine if a person is infected with HIV through their appearance?**		
Error/Unknown	*Ref*.	
Correct	0.55(0.42–0.70)	<0.001
**Should one proactively seek HIV counseling and testing after having high-risk sexual behavior?**		
Error/Unknown	*Ref*.	
Correct	0.60(0.40–0.90)	0.013
**Whether accepted the school’s publicity on AIDS testing in the last year**		
No	*Ref*.	
Yes	1.26(1.01–1.58)	0.039
**Whether accepted the AIDS risk self-assessment carried out by the school**		
No	*Ref*.	
Yes	1.64(1.32–2.04)	<0.001
**Whether accept to engage in one night stand behavior**		
Don’t accept/don’t know	*Ref*.	
Yes	3.23(2.53–4.12)	<0.001
**Whether accept to partake in commercial sexual activity**		
Don’t accept/don’t know	*Ref*.	
Yes	1.92(1.53–2.42)	<0.001
**Sexual partners**		
Heterosexual	*Ref*.	
Homosexual/Both	3.43(2.57–4.58)	<0.001
**Whether have been tested for HIV**		
No	*Ref*.	
Yes	1.36(1.02–1.82)	0.036
**Whether known that the CDC provides HIV testing**		
No	*Ref*.	
Yes	0.66(0.49–0.90)	0.008
**Demonstration of self-efficacy in condom usage** ^*^		
4 scores and below	*Ref*.	
5–8 scores	1.04(0.81–1.34)	0.759
9 scores	1.28(1.00–1.64)	0.048

^*^There is missing data.

^***#***^*CNY*, *Chinese yuan*.

## Discussion

We conducted a cross-sectional survey among universities in Zhejiang Province, providing valuable insights into the casual sexual behavioral characteristics of university students and the factors affecting these behaviors. The research revealed that self-reported and unsafe behaviors among university students are becoming increasingly common, indicating that high-risk behaviors are prevalent. Several studies have suggested a higher incidence of unsafe behaviors among college students due to the growing liberalization of sexual attitudes, thus creating a substantial risk for the transmission of HIV or STDs [16,19,20].

The results of this study indicated that college students engaged in casual sexual behavior displayed superior cognition on three knowledge-based questions. Our univariate analysis revealed slight variations in responses to the first question pertaining to male homosexuality between behavioral groups, although these differences were not statistically significant in the multivariate analyses. For questions two and three, the accuracy rates were greater for students who avoided casual sexual behavior. Prior studies have alluded to AIDS-related risky behavior being prevalent among the youth, with discrepancies noted between behavior and risk cognition related to HIV [21]. The current investigation determined that students engaged in casual behavior were 1.92 and 3.23 times more prone to indulging in commercial sex and accepting one-night stands, respectively, compared withstudents avoiding casual behavior. This is despite their superior knowledge of AIDS and more liberal attitudes toward sex. The incidence of unsafe high-risk behaviors was discernible. Earlier studies stressed raising AIDS awareness and fortifying consistency between acquired knowledge and subsequent action as key measures to protect susceptible groups, suggesting that future AIDS education for college students should prioritize consistency between knowledge, faith, and practice [22].

This study suggested that participants from urban areas, those with a monthly living cost less or equal to 1000 yuan, and male college students tended to have a higher frequency of casual sexual behaviours. Meanwhile, participants with a monthly living cost between 1001 and 1500 yuan and those originating from rural areas experienced a lower frequency of such behaviours. These findings align with previous conclusions, indicating a higher rate of sexual activity among urban and male college students [23]. Moderate monthly living expenses among students can help reduce the likelihood of casual sexual behaviours, which is an aspect worthy of attention.

The results of this study indicate that the proportion of college students in the casual sexual behavior group who had received school HIV testing publicity, self-assessment of HIV risks initiated by the school, and HIV antibody testing in the past year were all higher than those in the non-casual sexual behavior group. This shows that college students in the casual sexual behavior group had a certain degree of risk awareness. However, the proportion of individuals who underwent HIV antibody testing was only 10.6% (399/3771). At the same time, the sexual attitudes of the casual sexual behavior group were more open. Previous studies have shown that more openness to sexual attitudes is more likely to lead to unsafe behaviors, such as multiple sexual partners, presenting a higher risk of infection and sexual coercion [24]. HIV risk perception and testing awareness must be enhanced among college students. In future HIV prevention and education in colleges or universities, further emphasis should be placed on informed friendships, promoting voluntary testing, and reducing the threat of AIDS. Efforts to promote safe sexual behavior and create a good atmosphere to promote condom use and testing behavior are important strategies for preventing new infections [25].

The findings of this study imply that awareness of HIV antibody testing services provided by the CDC and having heterosexual sexual partners act as protective factors against casual risky sexual behaviors. On the other hand, scoring 9 on the condom use self-efficacy scale, compared with groups scoring less than 4, served as a risk factor for such behaviors. Remarkably, 90.1% (3396/3771) of the respondents were cognizant of the CDC’s HIV antibody testing services. Involvement in sexual behavior with homosexual partners may be linked to a heightened risk involving multiple sexual partners, which could increase the risk of disease transmission [15]. An investigation have determined the cognizance of the CDC’s HIV screening services, elevating HIV testing awareness, enhancing condom accessibility, promoting safer sexual behavior among university students, and mitigating the risk of HIV transmission [17]. Accordingly, HIV prevention and instructional efforts in schools should intensify collaboration with CDC professional agencies and universities, focus on promoting voluntary testing, augmenting condom accessibility, bolstering self-protection capabilities, and primarily focus on prevention and control among male students, especially within the homosexual community.

This study had several limitations. Given the cross-sectional survey structure, causal deductions regarding the identified factors remain speculative. The survey’s content relied heavily on respondents’ self-reports, contributing to possible biases. Crucial aspects such as the timing of initial sexual encounters and the use of protection in casual acts were absent among the study’s variables, potentially influencing the broad applicability of the findings. However, performing univariate and multivariate logistic regression analyses aimed reduce the impact of these limitations, thus providing research outcomes with some degree of scientific validity.

## Conclusions

In Zhejiang province, the proportions of sexual activity and casual sex among college students were comparatively high. Notably, there were greater percentages of male and urban-based students engaging in casual sex than their female and rural counterparts. These individuals tend to exhibit a liberal attitude toward sex coupled with a sense of HIV infection risk. However, their HIV testing rates were low, leading to knowledge-behavior discrepancies. Therefore, it is imperative to establish pertinent health education initiatives that align with this demographic characteristics, amplify anti-AIDS campaigns, underscore the function of disease control organizations, augment the facilitative environment for condom acquisition to increase its usage, and elevate testing rates and willingness to foster well-informed relationships. Such actions can help minimize the risk of HIV transmission among college students.

## Abbreviations

95% *CI*: 95% confidence interval
AOR: adjusted odds ratio
AIDS: Acquired Immune Deficiency Syndrome
HIV: Human immunodeficiency virus
STDs: Sexually transmitted diseases
CDC: Center for Disease Control and Prevention
CNY: Chinese yuan
